# A methodology for the public health surveillance and epidemiologic analysis of outdoor falls that require an emergency medical services response

**DOI:** 10.1186/s40621-023-00414-z

**Published:** 2023-01-12

**Authors:** Andrew G. Rundle, Remle P. Crowe, Henry E. Wang, Alexander X. Lo

**Affiliations:** 1 Department of Epidemiology, Columbia University Mailman School of Public Health, New York, NY 10032 USA; 2 ESO Solutions LLC, Austin, TX USA; 3 Department of Emergency Medicine, Wexner Medical Center, Ohio State University, Columbus, OH USA; 4 Department of Emergency Medicine, Northwestern University Feinberg School of Medicine, Chicago, IL USA; 5 Center for Health Services & Outcomes Research, Northwestern University Feinberg School of Medicine, Chicago, IL USA

**Keywords:** Falls, Injury, Outdoor falls, Surveillance

## Abstract

**Background:**

Falls are a common cause of injury with significantly associated medical costs yet public health surveillance of injuries from falls is underdeveloped. In addition, the epidemiologic understanding of outdoor falls, which have been reported to account for 47% of all injurious falls, is scant. Here we present methods to use emergency medical services (EMS) data as a public health surveillance tool for fall injuries, including those that occur secondary to syncope and heat illness, with a focus on the scope and epidemiology of outdoor fall injuries.

**Methods:**

Using the 2019 National Emergency Medical Services Information System (NEMSIS) data, we developed an approach to identify EMS encounters for fall injuries, syncope and heat illness. NEMSIS variables used in our algorithm included the EMS respondent’s impression of the encounter, the reported major symptoms and the cause of injury. With these data we identified injuries from falls and, using the NEMSIS data on the location of the encounter, we identified fall injuries as occurring indoors or outdoors. We present the descriptive epidemiology of the identified patients.

**Results:**

There were 1,854,909 injuries from falls that required an EMS response identified in the NEMSIS data, with 4% of those injuries secondary to episodes of syncope (*n* = 73,126) and heat illness. Sufficient data were available from 94% of injurious falls that they could be assigned to indoor or outdoor locations, with 9% of these fall injuries occurring outdoors. Among fall injuries identified as occurring outdoors, 85% occurred on streets and sidewalks. Patient age was the primary sociodemographic characteristic that varied by location of the injurious fall. Sixty-six percent of fall injuries that occurred indoors were among those age 65 years or older, while this figure was 34% for fall injuries occurring outdoors on a street or sidewalk.

**Conclusion:**

The occurrence of outdoor fall injuries identified in the NEMSIS data were substantially lower than reported in other data sets. However, numerically fall injuries occurring outdoors represent a substantial public health burden. The strengths and weaknesses of using this approach for routine public health surveillance of injuries from falls, syncope and heat illness are discussed.

## Background

The Centers for Disease Control and Prevention’s Web-based Injury Statistics Query and Reporting System reports that 7.9 million unintentional injurious falls occurred in 2019, with associated medical costs of 131.45 billion dollars (Centers for Disease Control and Prevention 2021). Falls are the leading cause of injuries and functional decline among older adults (Panel on Prevention of Falls in Older Persons et al. 2011). Outdoor falls have been reported to account for 47% to 58% of all falls, and up to a third of these outdoor falls are reported to have occurred on sidewalks, curbs and streets (Li et al. 2006; Timsina et al. 2017). Yet, outdoor falls are far less studied than indoor falls, with no proven mechanisms for public health surveillance and with distinct knowledge gaps on the role of urban design and environment, thus limiting the development of community-specific and person-centered prevention programs (Chippendale et al. 2017; Li et al. 2006). Unlike indoor falls, which are more likely influenced by an individual's risk factors and health status, outdoor falls are often associated with health-promoting activities (e.g., walking, running, gardening) instead of poor health and are, in particular, substantially influenced by environmental factors, climate (e.g., winter weather, rain and extreme heat) and the physical aspects of the environment such as poorly maintained paving (Chippendale et al. Kelsey et al. 2012a, 2012b; Li et al. 2006; Satariano et al. 20172017). While icy conditions are a known cause of outdoor falls, there is emerging evidence that injurious outdoor falls are also associated with higher temperatures (Lee et al. 2022; Vongsachang et al. 2021).

The primary public health surveillance tools for fall-related injuries are the Behavioral Risk Factor Surveillance System (BRFSS) and the National Health Interview Survey (NHIS), both of which collect self-reported data via telephone surveys (Moreland et al. 2020; Timsina et al. 2017). The BRFSS does not collect data on falls annually and does not distinguish falls by location (Moreland et al. 2020). The NHIS can be used to identify falls that occur indoors or outdoors, but the location data reside in the unstructured narrative data which are cumbersome to analyze (Timsina et al. 2017). A critical first step to developing effective interventions to reduce outdoor falls is to have efficiently generated, robust and reliable surveillance and epidemiological data to characterize the burden of falls, identify high-risk populations, and monitor the distribution of this burden across place and time. Location and temporal differences in fall rates provide the optimal means to examine the role of the environmental, seasonal, temperature, climate and built environment risk factors for outdoor falls (Morency et al. 2012; Qian et al. 2019; Vongsachang et al. 2021).

In conceptualizing an epidemiologic surveillance strategy for falls, it may be important to also examine syncope and heat-related illnesses. These latter conditions not only contribute to injurious falls but are also misclassified as falls and may influence the validity of case ascertainment and measurement of falls. (Anpalahan 2006; Lo et al. 2022; O'Mahony et al. 1998; Rubenstein et al. 2002; Shaw et al. 1997; Ungar et al. 2016). This is particularly an issue among older adults, where the clinical spectrum of falls and syncope overlap significantly (Anpalahan 2006; Lo et al. 2022; O'Mahony et al. 1998). In addition, the epidemiologic characteristics of fall injuries occurring secondary to syncope and heat illness may differ from those for mechanical falls.

Here we present an algorithm to identify injurious falls overall, and those secondary to episodes of syncope and heat illness, and injurious falls by indoor versus outdoor location, using EMS clinical and administrative data from the National Emergency Medical Services Information System (NEMSIS).

## Methods

For this retrospective study we used the 2019 NEMSIS data set which is a national, publicly available database that has standardized the collection and aggregation of deidentified information on EMS care in the USA and is a product of the National Highway Traffic Safety Administration Office of Emergency Medical Services (Dawson 2006; Hanlin et al. 2022). We did not use more recent versions of the NEMSIS in order to avoid the impact of the COVID-19 pandemic. Entries in NEMSIS were excluded if the Disposition of the response was listed as Canceled (codes 4212007, 4212009, 4212011), Standby-No Services or Support Provided (code 4212039), or Transport Non-patient (code 4212043) or eResponse was coded as Interfacility Transport (code 2205003) or Medical Transport (code 2205007).

### Identifying injuries from falls and episodes of syncope and heat-related illness in EMS administrative data

The EMS respondent’s judgement on the cause of any injury suffered by the patient is documented via ICD10 codes in the NEMSIS eInjury.01 variable. Two of us (AGR and AXL) reviewed the ICD codes included in the eInjury.01 variable to select codes indicating an injury due to a fall. Given the clinical overlap between falls, syncope and heat illness, and because fall injuries that occur secondary to syncope and heat illness may have risk factors that differ from those for mechanical falls, we also sought to create a classification schema to identify EMS encounters for syncope and heat illness and to identify fall injuries occurring secondary to syncope or heat illness.

The EMS responder’s overall impression of the encounter is documented via ICD10 codes in the eSituation.11 and eSituation.12 variables. We coded EMS responses as being for “Syncope without a heat component” if eSituation.11 or eSituation.12 contained an ICD 10 code for syncope and neither variable included an ICD 10 coded for heat-related encounters. We coded EMS responses as being for “Syncope with a heat component” if eSituation.11 or eSituation.12 contained an ICD 10 code for syncope or heat syncope and either variable included an ICD 10 coded for heat-related encounters. We coded EMS responses as being for “Heat related illness—no syncope” if eSituation.11 or eSituation.12 contained an ICD 10 code for a heat encounter other than Heat Cramps and neither variable included an ICD 10 code for syncope. This classification created four mutually exclusive categories, the fourth being an EMS response to an encounter other than for syncope or heat-related illness.

Among patients whose encounter was not classified as syncope with or without a heat component and not classified as “Heat related issue with no syncope” via the eSituation.11 and eSituation.12 variables, we also sought to classify encounters as belonging to one of these three categories using the EMS clinician’s report of the primary symptom, which is documented in the eSituation.09 variable. Upon inspection of instances where eSituation.09 included syncope or heat-related ICD 10 codes, combinations of ICD 10 codes reported in eSituation.09, eSituation.11 and eSituation.12 were selected that likely indicated syncope and heat-related encounters. If eSituation.09 included an ICD 10 code for syncope and eSituation.11 reported ICD 10 codes for weakness, dizziness and giddiness, hypotension or altered mental state and eSituation.12 did not include a code for heat-related encounters, the encounter was coded as “Syncope without a heat component”. If eSituation.09 was coded as syncope and eSituation.11 was coded as dehydration, the compliant was coded as “Syncope with a heat component”. If eSituation.09 was coded as heat-related and eSituation.11 was coded as weakness, dizziness and giddiness, hypotension or altered mental state and eSituation.12 did not include a code for syncope, the encounter was coded as “Heat related issue with no syncope”.

To estimate the contribution of syncope and heat-related illness to injuries from falls, we cross-classified EMS responses by the “Injury from Fall” variable derived from eInjury.01 with the “Syncope without a heat component”, “Syncope with a heat component” and “Heat related illness—no syncope” variable. This cross-classification created four mutually exclusive EMS response categories: (1) “Syncope without a heat component”; (2) “Syncope with a heat component”; (3) “Heat related illness—no syncope”; and 4) “Injury from Fall without a syncope or heat component”. The cross-classification also allowed for sub-categorization of categories 1, 2 and 3, as including a fall related injury and no fall related injury.

Because seizures can result in injuries from falling we also sought to exclude instances of seizure. If eSituation.09, eSituation.11 or eSituation.12 included a coded for seizure (G56 and subcodes, G40 and subcodes or F44.5) the patient was excluded from the analyses of syncope, heat or fall related encounters.

### Location of EMS encounter

Data from the NEMSIS eScene.09 variable was used to code the patient locations as “Indoors”, “Outdoors—not on a street or sidewalk”, “Outdoors—on a street or sidewalk” and “Not on a street, but Indoors vs. Outdoors not Definable”. The eInjury.01 ICD 10 codes can also provide information on the location of the patient at the time of the encounter (e.g., fall from toilet suggests an indoor location). When the patient location could not be classified using the eScene.09 variable, any available information from the eInjury.01 variable was used to assign a location.

### Sociodemographic data

Data from NEMSIS variables were used to categorize the age (age years), sex (ePatient.13), race and ethnicity (ePatient.14) of the patient. The CensusDivision variable was used to define the region of the USA in which the incident occurred and the Census Divisions were coded as South, North and West; the Pacific and Mountain regions stretch from the Northern to the Southern border of the USA and so were grouped together as the West.

### Statistical analyses

Descriptive analyses were conducted of the EMS encounters for “Syncope without a heat component”, “Syncope with a heat component”, “Heat related illness—no syncope” and “Injury from Fall without a syncope or heat component”. Likewise descriptive analyses were conducted of EMS encounters for injuries from falls.

## Results

The 2019 NEMSIS data set included 34,203,087 records of EMS responses, of which 33% met either of the Disposition or eResponse-based exclusion criteria, leaving 23,086,855 EMS encounters where patient care was provided. Of the 23,086,855 EMS encounters there were 2,526,870 that were classified into one of the four fall, syncope, or heat-related issue categories. Of these, there were 54,738 (2%) encounters that included a seizure-related code and were excluded. Thus in total, there were 2,472,132 encounters for fall injuries, syncope or heat-related issues that met the inclusion criteria: 1,780,371 for “Injury from Fall without a syncope or heat component”; 643,260 for “Syncope without a heat component”; 6550 for “Syncope with a heat component”; and 41,951 for “Heat related illness—no syncope”. In total there were 1,854,909 fall injuries, including: 73,126 from the “Syncope without a heat component” category; 474 from the “Syncope with a heat component” category; and 938 from the “Heat related illness—no syncope” category.

Table 1 reports on the location where the EMS clinician encountered the patient. It was not possible to assign the encounter to an indoor or outdoor location for 3% of the encounters, for example, an encounter for a fall reported to have occurred on a tennis court might have occurred indoors or outdoors. For another 3% of encounters a location was not reported. Of the encounters where a location could be defined, 9% were reported as occurring outdoors, with 147,225 outdoor encounters for “Injury from Fall without a syncope or heat component”, and 48,475 outdoor encounters for “Syncope without a heat component”, 1084 outdoor encounters for “Syncope with a heat component”, and 11,246 outdoor encounters for “Heat related illness—no syncope”. In total, 85% of the patients who were identified as being encountered outdoors were encountered on streets or sidewalks. Table 1 also reports on the social demographic characteristics of the patients classified into each of the four encounter categories.

**Table 1:** EMS Encounters for Falls, Syncope with and without a heat component and heat illness

	Fall injury without syncope or heat *N* (%)	Syncope–no heat component *N* (%)	Syncope–with heat component *N* (%)	Heat-related issue but no syncope *N* (%)
Total N	1,780,371	643,260	6550	41,951
*Reported Injury*				
No injury or injury other than fall related	NA	570,134 (89)	6076 (93)	41,013 (98)
Injury from a fall	1,780,371 (100)	73,126 (11)	474 (7)	938 (2)
*EMS reported location of the patient*				
Indoors	1,531,903 (86)	545,846 (85)	4,948 (76)	26,411 (63)
Outdoors, not on a street or sidewalk	22,732 (1)	7539 (1)	446 (7)	3094 (7)
Outdoors, on a street or sidewalk	124,493 (7)	40,936 (6)	638 (10)	8152 (19)
Indeterminant for indoor vs outdoor status	51,307 (3)	22,896 (4)	406 (6)	3120 (7)
Not Recorded	49,936 (3)	26,043 (4)	112 (2)	1174 (3)
*Age Group*				
< 21	111,710 (6)	59,332 (9)	951 (15)	6107 (15)
21–29	57,667 (3)	50,243 (8)	661 (10)	5208 (12)
30–39	75,811 (4)	49,778 (8)	583 (9)	5483 (13)
40–49	93,113 (5)	53,949 (8)	570 (9)	5026 (12)
50–64	308,128 (17)	128,403 (20)	1378 (21)	9406 (22)
65 +	1,128,394 (63)	299,049 (46)	2394 (37)	10,575 (25)
Not Recorded	5548 (< 1)	2506 (< 1)	13 (< 1)	146 (< 1)
*Sex of patient*				
Male	742,993 (42)	297,791 (46)	3465 (53)	26,777 (64)
Female	1,030,141 (58)	342,114 (53)	3,054 (47)	14,954 (36)
Not Recorded	7237 (< 1)	3355 (1)	31 (< 1)	220 (1)
*Race and Ethnicity*				
American Indian or Alaska Native	8011 (< 1)	2827 (< 1)	24 (< 1)	232 (1)
Asian	11,568 (1)	4132 (1)	41 (1)	331 (1)
Black or African American	193,587 (11)	70,923 (11)	779 (12)	5,280 (13)
Hispanic or Latin	68,518 (4)	24,949 (4)	274 (4)	1727 (4)
Native Hawaiian or Other Pacific Islander	2463 (< 1)	935 (< 1)	16 (< 1)	64 (< 1)
White	548,779 (31)	198,113 (31)	2093 (32)	14,232 (34)
Mixed Race	1218 (< 1)	459 (< 1)	3 (< 1)	42 (< 1)
Not Recorded	946,227 (53)	340,922 (53)	3320 (51)	20,043 (48)
*South, North or West Census Divisions*				
North	502,155 (28)	179,967 (28)	1525 (23)	8363 (20)
South	806,320 (45)	285,314 (44)	3495 (53)	25,155 (60)
Pacific or Mountain	471,459 (26)	177,516 (28)	1528 (23)	8424 (20)
Not Recorded	437 (< 1)	463 (< 1)	2 (< 1)	9 (< 1)
*Urbanicity*				
Urban	1,482,677 (83)	546,442 (85)	5,042 (77)	33,672 (80)
Suburban	97,037 (5)	30,644 (5)	438 (7)	2476 (6)
Rural	117,281 (7)	36,968 (6)	628 (10)	3419 (8)
Wilderness	31,894 (2)	9455 (1)	164 (3)	747 (2)
Not Recorded	51,482 (3)	19,751 (3)	278 (4)	1637 (4)

Table 2 compares the characteristics of patient encounters for “Injury from Fall without a syncope or heat component” and for fall injuries secondary to “Syncope without a heat component”. Patient age and sex were the only sociodemographic variable that meaningfully varied by these two fall categories. Among encounters for “Injury from Fall without a syncope or heat component” 63% of patients were age 65 years or older, while for fall injuries secondary to “Syncope without a heat component” only 48% of patients were in this age group. Female patients predominated (58%) among encounters for “Injury from Fall without a syncope or heat component”, while patient encounters for fall injuries secondary to “Syncope without a heat component” were essentially evenly divided between male and female patients.

**Table 2:** Fall injuries without syncope or heat component versus fall injuries secondary to Syncope without Heat Component

	Fall injury without Syncope or Heat *N* (%)	Fall injury secondary to Syncope–no heat component *N* (%)
N	1,780,371	73,126
*Location reported by EMS*		
Indoors	1,531,903 (86)	63,975 (87)
Outdoors, not on a street or sidewalk	22,732 (1)	762 (1)
Outdoors, on a street or sidewalk	124,493 (7)	4672 (6)
Indeterminant for indoor vs outdoor status	51,307 (3)	2323 (3)
Not Recorded	49,936 (3)	1394 (2)
*Age Group*		
< 21	111,710 (6)	6284 (9)
21–29	57,667 (3)	5189 (7)
30–39	75,811 (4)	5145 (7)
40–49	93,113 (5)	6049 (8)
50–64	308,128 (17)	15,205 (21)
65 +	1,128,394 (63)	35,121 (48)
Not Recorded	5548 (< 1)	133 (< 1)
*Sex of patient*		
Male	742,993 (42)	36,148 (49)
Female	1,030,141 (58)	36,762 (50)
Not Recorded	7237 (< 1)	216 (< 1)
*Race and Ethnicity*		
American Indian or Alaska Native	8011 (< 1)	296 (< 1)
Asian	11,568 (1)	470 (1)
Black or African American	193,587 (11)	7972 (11)
Hispanic or Latin	68,518 (4)	2761 (4)
Native Hawaiian or Other Pacific Islander	2463 (< 1)	122 (< 1)
White	548,779 (31)	22,654 (31)
Mixed Race	1218 (< 1)	51 (< 1)
Not Recorded	946,227 (53)	38,800 (53)
*South, North or West Census Divisions*		
North	502,155 (28)	18,952 (26)
South	806,320 (45)	31,734 (43)
Pacific or Mountain	471,459 (26)	22,421 (31)
Not Recorded	437 (< 1)	19 (< 1)
*Urbanicity*		
Urban	1,482,677 (83)	62,898 (86)
Suburban	97,037 (5)	3314 (5)
Rural	117,281 (7)	3802 (5)
Wilderness	31,894 (2)	1040 (1)
Not Recorded	51,482 (3)	2072 (3)

Table 3 reports on the sociodemographic characteristics of all fall injuries by reported location of the encounter. Ninety-four percent of fall injuries could be classified as to occurring at an indoor or outdoor location, with 91% of these fall injuries occurring indoors. Among fall injuries identified as occurring outdoors, 85% occurred on streets and sidewalks. Patient age was the primary sociodemographic variable that varied by fall injury location category. Among fall injuries occurring indoors, 66% occurred among patients age 65 years or older in age, while for fall injuries occurring outdoors, not on streets or sidewalks, 26% occurred in this age group and for fall injuries occurring outdoors on a street or sidewalk 34% occurred in this age group. For the two outdoor fall injury categories around 50% occurred among those age 50 years or older; 45% for fall injuries occurring outdoors, not on a street or sidewalk and 62% for fall injuries occurring outdoors on a street or sidewalk.

**Table 3:** Descriptive Data for EMS Encounters for Fall Injuries by EMS-Reported Location

	EMS reported location of the patient				
	Indoors	Outdoors, not on a street or sidewalk *N* (%)	Outdoors, on a street or sidewalk *N* (%)	Indeterminant for indoor vs. outdoor status *N* (%)	Not recorded *N* (%)
Total N	1,596,860	23,586	129,408	53,700	51,355
*Age Group*					
< 21	90,329 (6)	6835 (29)	8998 (7)	9061 (17)	2916 (6)
21–29	44,162 (3)	1924 (8)	11,188 (9)	4148 (8)	1542 (3)
30–39	59,163 (4)	2025 (9)	13,544 (10)	4418 (8)	1919 (4)
40–49	76,151 (5)	2066 (9)	14,181 (11)	4595 (9)	2273 (4)
50–64	263,794 (17)	4414 (19)	36,433 (28)	11,607 (22)	7405 (14)
65 +	1,058,803 (66)	6233 (26)	44,286 (34)	19,756 (37)	35,056 (68)
Not Recorded	4458 (< 1)	89 (< 1)	778 (1)	115 (< 1)	244 (< 1)
*Sex of patient*					
Male	644,727 (40)	12,413 (53)	74,707 (58)	26,950 (50)	21,222 (41)
Female	946,122 (59)	11,067 (47)	54,077 (42)	26,525 (49)	29,639 (58)
Not Recorded	6011 (< 1)	106 (< 1)	624 (< 1)	225 (< 1)	494 (1)
*Race and ethnicity of patient*					
American Indian or Alaska Native	7163 (< 1)	83 (< 1)	601 (< 1)	261 (< 1)	201 (< 1)
Asian	10,363 (1)	164 (1)	898 (1)	330 (1)	295 (1)
Black or African American	174,202 (11)	2568 (11)	14,188 (11)	5832 (11)	4951 (10)
Hispanic or Latin	61,489 (4)	958 (4)	5024 (4)	2105 (4)	1768 (3)
Native Hawaiian or Other Pacific Islander	2229 (< 1)	35 (< 1)	169 (< 1)	80 (< 1)	78 (< 1)
White	493,615 (31)	7262 (31)	40,278 (31)	15,959 (30)	14,773 (29)
Mixed Race	1103 (< 1)	19 (< 1)	73 (< 1)	48 (< 1)	27 (< 1)
Not Recorded	846,696 (53)	12,497 (53)	68,177 (53)	29,085 (54)	29,262 (57)
*South, North or West Census Divisions*					
North	441,323 (28)	7428 (31)	35,878 (28)	25,824 (48)	10,977 (21)
South	730,329 (46)	10,746 (46)	51,076 (39)	13,826 (26)	32,832 (64)
Pacific or Mountain	424,827 (27)	,395 (23)	42,429 (33)	14,041 (26)	7522 (15)
Not Recorded	381 (< 1)	17 (< 1)	25 (< 1)	9 (< 1)	24 (< 1)
*Urbanicity*					
Urban	1,320,032 (83)	18,514 (78)	114,541 (89)	47,269 (88)	46,307 (90)
Suburban	90,674 (6)	1349 (6)	4719 (4)	1783 (3)	1927 (4)
Rural	109,808 (7)	2127 (9)	5342 (4)	2328 (4)	1606 (3)
Wilderness	29,453 (2)	700 (3)	1600 (1)	997 (2)	223 (< 1)
Not Recorded	46,893 (3)	896 (4)	3206 (2)	1323 (2)	1292 (3)

## Discussion

These data demonstrate the annual burden of injurious falls in the USA receiving treatment by EMS clinicians, and the contribution of syncope and heat illness to the burden of fall injuries. Our work provides an approach for surveillance of these health outcomes using routinely collected EMS administrative and clinical data. Ongoing analysis of EMS administrative and clinical data could identify sociodemographic, temporal, regional and neighborhood clusters of injuries from falls that signal where cities could invest to improve infrastructure to reduce injurious falls. In addition, this approach can be used to create analytical data sets for research studies, such as ecological or location-based case–control studies, to identify risk factors for fall injuries or demonstrate the impact of interventions to reduce injurious falls (Mooney et al. 2022).

The National Health Interview Survey (NHIS) provides national data on falls and the NHIS narrative responses can be analyzed to identify the location where the injurious fall occurred (Timsina et al. 2017). Analyses of the 1997 to 2010 NHIS data found that 47% of fall related injuries requiring medical attention among community dwelling adults occurred outdoors (Timsina et al. 2017). Of these injuries, 6.5%, an estimated 518,000 fall injuries annually, occurred on sidewalks, curbs or streets. However, these NHIS data are now 10–25 years old and, because of the intensive coding of free text required in the methods, it is difficult to update these analyses using NHIS (Timsina et al. 2017). The estimate of the percent of injurious falls that occur outdoors from NHIS data is substantially higher than observed in the NEMSIS data. However, the NHIS collects data from community dwelling adults, while NEMSIS reflects all EMS responses including those to senior living facilities where falls may be more likely to occur indoors. Furthermore, the NHIS included injurious falls that did and did not require an immediate EMS response. Since outdoor falls generally occur among individuals healthy enough to venture outdoors, it is possible that injurious falls occurring outdoors are less likely to require an EMS response than those occurring indoors.

We identified 129,408 fall injuries requiring an EMS response that occurred outdoors on streets and sidewalks. Placing this in context, in 2019 this figure is 70% higher than estimates of the number of pedestrians (76,000) injured by automobiles in 2019 (National Highway Traffic Safety Administration 2021). While there is a large body of research on pedestrian injuries from automobiles (Halari et al. 2020; Namatovu et al. 2022; Rezapur-Shahkolai et al. 2022; Wilmut et al. 2022), there is almost no literature on pedestrian injuries from falls that occur on streets and sidewalks (Timsina et al. 2017). This discrepancy in research attention translates into a marked focus on policy and design interventions to prevent pedestrian injuries from automobiles, including the national Vison Zero program, and minimal focus on interventions to prevent pedestrian injuries from outdoor falls, even though these injuries occur in the same or adjacent physical environments (Evenson et al. 2018).

While little is known about environmental risk factors for outdoor falls, icy conditions and uneven pavements are known proximal causes of pedestrian falls and a difference in rise between sidewalk pavers as small as 1.5 cm is sufficient to pose a risk (Bentley 1998, 2001; Curl et al. 2016; David et al. 1990; Fothergill et al. 1995; Hunt et al. 1991; Timsina et al. 2017) In many cities the burden of sidewalk maintenance falls entirely or partially on the property owner whose land abuts the sidewalk, although some cities have programs to help property owners fix the sidewalks (City of Minneapolis 2021; City of New York 2019; Downtown Memphis Commission 2020; McPherson 2000; Rae et al. 2011). Because the burden of maintenance is on individual property owners the physical quality of the sidewalk and extent of snow and ice removal along a single block can vary widely. Routine surveillance for outdoor fall injuries, along with options for pedestrians to report sidewalk damage, could be used to target city enforcement of sidewalk maintenance or target funds to support property owners in maintaining the sidewalks (City of Minneapolis 2021; City of New York 2019; Downtown Memphis Commission 2020).

Street tree planting campaigns are a common component of urban greening and climate change mitigation efforts and may reduce heat-related health encounters, but are also associated with sidewalk damage (McPherson 2000; Mullaney et al. 2015; Rae et al. 2011; Vogt et al. 2015). Best practices guidelines for planting street trees, and for ongoing tree maintenance, to minimize sidewalk damage have been published (MillionTreesNYC 2015; Mullaney et al. 2015).Although sidewalk damage from street trees may increase risk for falls, the cooling effects of trees may prevent some falls. A study among individuals at high risk of falling found that higher temperatures were associated with significantly higher odds of a fall being injurious, as opposed to non-injurious (Vongsachang et al. 2021). An ecologic study of Census blocks in Marin County CA found that a higher extent of tree canopy cover over streets was associated with lower rates of pedestrian falls among adults age 65 and older (Lee et al. 2022). Because this association was only found during periods when leaves were on the trees (leaf on months), the authors suggest that shade-related lowering of local ambient temperatures may explain the reduction in fall injuries (Lee et al. 2022). As syncope and heat illness are related to high temperatures, lower ambient temperatures may also reduce outdoor fall injuries occurring secondary to syncope and heat illness (Heo et al. 2021; Horváthová et al. 2021; Kilbourne et al. 1982). The trade-offs between the possibility of increased injuries from trip and fall events related to street trees damaging sidewalks and the occurrence of fewer syncope and heat illness events due to trees reducing local temperatures have not been studied.

Some strengths and weaknesses of the approach described here should be noted, the primary strength being that NEMSIS provides a well-documented nationally representative census of health encounters requiring an EMS response. The case-identification strategy relies on limited ICD 10 codes entered into specific data fields by EMS responders. Much of the data is supplied to NEMSIS by third-party companies that provide data services to EMS companies and health care systems. The data entry systems provided by these third-party companies vary and this may influence the coding available to EMS personnel and thus data quality may vary by provider and between regions these companies operate in. However, these private companies could begin selling “public health surveillance as service” data products and develop a sustainable model for such analyses. Another limitation is that the coding schema does not make use of narratives and text notes created by EMS personnel. As such, the reliance on ICD 10 codes may result in an undercount of encounters that included syncope, heat-related issues and fall injuries. Prior analyses of the 1997–2010 NHIS used data coding of free text which could also be applied to EMS narrative notes, but such work is labor intensive. However, machine learning for natural language processing applied to EMS narrative notes could supplement the ICD 10-based case-finding algorithm and increase the sensitivity of identifying these encounters from EMS data. Further research is needed to demonstrate the feasibility of automated processing of EMS notes to identify syncope, heat-related issues and injuries from falls and determine whether increased sensitivity comes at the cost of reduced specificity.

## Conclusions

Our work provides an approach for health surveillance for injurious falls, syncope and heat illness, using routinely collected EMS administrative data, with the benefit of being able to attribute these events to indoor or outdoor locations. These health outcomes represent significant public health, clinical and economic burdens which are likely to increase with climate change and increasing temperatures. These burdens and their likelihood of increasing in the coming years emphasizes the need for efficient, routine health surveillance tools.

## Data Availability

The data sets analyzed during the current study are publicly available at https://nemsis.org.

## Abbreviations

BRFSS: Behavioral Risk Factor Surveillance System
EMS: Emergency Medical Services
ICD 10: International Classification of Diseases 10th Revision
NHIS: National Health Interview Survey
NEMSIS: National Emergency Medical Services Information System
