# Correction: A methodology for the public health surveillance and epidemiologic analysis of outdoor falls that require an emergency medical services response

**DOI:** 10.1186/s40621-023-00469-y

**Published:** 2023-11-03

**Authors:** Andrew G. Rundle, Remle P. Crowe, Henry E. Wang, Alexander X. Lo

**Affiliations:** 1 Department of Epidemiology, Columbia University Mailman School of Public Health, New York, NY 10032 USA; 2 ESO Solutions LLC, Austin, TX USA; 3 Department of Emergency Medicine, Wexner Medical Center, Ohio State University, Columbus, OH USA; 4 Department of Emergency Medicine, Northwestern University Feinberg School of Medicine, Chicago, IL USA; 5 Center for Health Services & Outcomes Research, Northwestern University Feinberg School of Medicine, Chicago, IL USA

**Correction: Injury Epidemiology (2023) 10:4** 10.1186/s40621-023-00414-z

Following publication of the original article (Rundle et al. 2023), the authors identified an error in the Funding section which lacked that Alexander X. Lo was supported by a grant from the Davee Foundation (Excellence in Emergency Medicine Grant).

The Funding section currently reads:

**Funding**

Not applicable.

The Funding section should read:

**Funding**

Dr. Lo was supported by a grant from the Davee Foundation (Excellence in Emergency Medicine Grant).

The Funding section has been updated in this correction and the original article (Rundle et al. 2023) has been corrected.
